# Vasectomy surgical techniques in South and South East Asia

**DOI:** 10.1186/1471-2490-5-10

**Published:** 2005-05-25

**Authors:** Michel Labrecque, John Pile, David Sokal, Ramachandra CM Kaza, Mizanur Rahman, SS Bodh, Jeewan Bhattarai, Ganesh D Bhatt, Tika Man Vaidya

**Affiliations:** 1 Department of Family Medicine, Laval University, Quebec City, Canada; 2 EngenderHealth, 440 Ninth Avenue, New York, NY 10001 USA; 3 Family Health International, 2224 Chapel Hill-Nelson Hwy Durham, NC, 27713 USA; 4 Maulana Azad Medical College, New Delhi, India; 5 EngenderHealth, Bangladesh Country Office, Dhaka, Bangladesh; 6 EngenderHealth, India Country Office, New Delhi, India; 7 Chhetrapati Family Welfare Center, Chhetrapati, Kathmandu Nepal; 8 Nepal Fertility Care Center, Jwagal Kopundole, Laitpur Nepal

## Abstract

**Background:**

Simple ligation of the vas with suture material and excision of a small vas segment is believed to be the most common vasectomy occlusion technique performed in low-resource settings. Ligation and excision (LE) is associated with a risk of occlusion and contraceptive failure which can be reduced by performing fascial interposition (FI) along with LE. Combining FI with intra luminal thermal cautery could be even more effective. The objective of this study was to determine the surgical vasectomy techniques currently used in five Asian countries and to evaluate the facilitating and limiting factors to introduction and assessment of FI and thermal cautery in these countries.

**Methods:**

Between December 2003 and February 2004, 3 to 6 major vasectomy centers from Cambodia, Thailand, India, Nepal, and Bangladesh were visited and interviews with 5 to 11 key informants in each country were conducted. Vasectomy techniques performed in each center were observed. Vasectomy techniques using hand-held, battery-driven cautery devices and FI were demonstrated and performed under supervision by local providers. Information about interest and open-mindedness regarding the use of thermal cautery and/or FI was gathered.

**Results:**

The use of vasectomy was marginal in Thailand and Cambodia. In India, Nepal, and Bangladesh, vasectomy was supported by national reproductive health programs. Most vasectomies were performed using the No-Scalpel Vasectomy (NSV) technique and simple LE. The addition of FI to LE, although largely known, was seldom performed. The main reasons reported were: 1) insufficient surgical skills, 2) time needed to perform the technique, and 3) technique not being mandatory according to country standards. Thermal cautery devices for vasectomy were not available in any selected countries. Pilot hands-on assessment showed that the technique could be safely and effectively performed by Asian providers. However, in addition to provision of supplies, introducing cautery with FI could be associated with the same barriers encountered when introducing FI in combination with LE.

**Conclusion:**

Further studies assessing the effectiveness, safety, and feasibility of implementation are needed before thermal cautery combined with FI is introduced in Asia on a large scale. Until thermal cautery is introduced in a country, vasectomy providers should practice LE with FI to maximize effectiveness of vasectomy procedure.

## Background

Vasectomy is recognized as a simple, safe, and effective contraception method. However, the occlusive and contraceptive effectiveness of the procedure varies widely according to the surgical technique used to occlude the vas deferens.[1] Ligation with suture material and excision of a small vas segment is believed to be the most common method used world-wide.[2] The risk of occlusive failure with this technique has been traditionally considered to be in the order of 1% to 5%.[3] However, recent studies have shown that the risk could be much higher, ranging from 8% to 13%, based on data from semen analyses.[4,5] The risk of contraceptive failure may also be unacceptably high. A study involving 1052 men in Nepal showed that within 3 years after vasectomy 4.2 % had an unplanned pregnancy.[6] A similar failure rate (4.1%) was also found in Vietnam after more than 5 years of follow-up.[7] In a study conducted in China, among 1,555 couples using vasectomy as a contraceptive method, the risk an unplanned pregnancy was 9.5% after 5 years.[8]

Alerted by this information Family Health International (FHI) and EngenderHealth, two non-governmental organizations promoting best practices in family planning worldwide, initiated an international research program to evaluate the effectiveness of alternative surgical techniques to LE. They recently completed two major studies.

The first was a multicenter randomized controlled trial (RCT) comparing occlusion by suture LE with versus without fascial interposition (FI). The interim analysis showed a clear advantage of FI and recruitment was halted in May 2001.[9] At that time 841 men had been enrolled. Final results from this cohort were published recently.[10] Using a definition of failure as > 5 million motile sperm / mL at 14 weeks or later or the presence more than 100,000 sperm with any motility at 26 weeks or later, they found 24 (5.9%) failures in the FI group versus 53 (12.7%) in the non-FI group. Most of the failures appeared to be due to early recanalization. [10]

Given the results of the RCT showing relatively high failure rates even with FI, the second study, an exploratory observational study of cautery of the vas lumen,[11] was initiated based on the recommendations from the Expert Consultation on Vasectomy Effectiveness, a meeting organized by FHI and EngenderHealth.[12] This study was conducted at four centers that routinely used cautery for vas occlusion. Frequency of semen analyses and laboratory procedures were similar in both the cautery study and the RCT, but follow-up was only through 24 weeks and some of the sites could not provide data on sperm motility. Using a definition of early failure as >10 million sperm / mL at 12 weeks regardless of motility, the risk of early failures was 4/389 (1.0%). Applying the same definition of failure to the RCT data set, early occlusive failure risks were 4.9% and 12.5% in the groups with and without FI, respectively.[13]

Though the results are encouraging for the use of cautery in vasectomy, they must be interpreted with caution based on this non-randomized comparison. In addition, while FI was showed to be important in improving vasectomy occlusion success when LE are the primary occlusion method, this exploratory study of cautery cannot definitively confirm that FI is as useful when cautery is used as the primary occlusion method. However, these findings support the results from numerous large case series showing that the occlusive effectiveness of cautery, especially when combined with FI on the prostatic end, is high, with failures well below 1%. [1,14-21]

In December 2003, FHI and EngenderHealth organized a three-day expert consultation on vasectomy techniques and services (Summary available on FHI's web site at ). The vasectomy experts recommended that 1) training of vasectomy providers emphasize the potential increased effectiveness of vasectomy when FI is added to the standard technique of LE; 2) providers now using simple LE consider adopting FI, with appropriate training as needed; 3) where resources, training, and logistical support are available, cautery can be considered as an effective and safe method to block the vas.

FI and/or cautery are already widely used in developed countries.[22] This might not be the case in developing countries[2] but there are no good data on the specific techniques used at the level of one country or a region in the developing world. It is just recently, in the 2003 edition of *No-scalpel vasectomy: An illustrated guide for surgeons*, that EngenderHealth has started to promote FI along with LE as the preferred occlusion technique, and cautery, with or without FI, as the alternative.[23] The rapid adoption of the most effective vasectomy occlusion techniques is essential considering that the lack of resources in most developing countries precludes most men from verifying the success of their vasectomy with semen analysis. EngenderHealth currently recommends that men use another form of contraception during the first 12 weeks after vasectomy.

However, many barriers could prevent the adoption of these techniques in the low-resource settings. Firstly, while "low tech" hand-held battery-driven thermal cautery devices are available, the instruments and supplies needed to perform cautery and FI (cautery device, tips, batteries, and suture material) may be difficult to procure, to use properly – including to sterilize adequately – and to maintain for further use. Secondly, performing cautery and FI require additional surgical skills over LE. Specific hands-on training is essential to master these techniques although cautery is much easier to perform adequately than FI. Thirdly, personal, professional, social constraints, or country standards may limit the adoption of new surgical techniques, especially if the changes are imposed without adequate scientific justification and appropriate training.

Accurate information on the current situation is essential to evaluate these barriers and to successfully introduce and adequately evaluate the adoption of new vasectomy occlusion techniques. This paper reports on the vasectomy techniques currently used in major vasectomy clinics or programs in Cambodia, Thailand, India, Nepal, and Bangladesh, and on the factors that could facilitate or obstruct the introduction of vasectomy occlusion techniques using cautery and FI in these countries.

## Methods

Countries were selected based on the involvement of national or international organizations supporting family planning programs in each of the countries, and geographical proximity to each other. These countries represent a wide range of cultural and religious backgrounds facilitating the generalization of the results. In all the selected countries, the use of vasectomy is currently much lower than the use of tubal ligation (Table 1). This represents a window of opportunity in most of these countries for promoting best practices related to male sterilization and to enhance the popularity of vasectomy.

**Table 1:** Proportion of women currently married or in union using sterilization as a family planning method in selected Asian countries.

**Countries**	**Using Male Sterilization %**	**Using Female sterilization %**	**Year(s) of Assessment**
**Cambodia**	-	1.5	2000
**Thailand**	2.0	22.0	1996–7
**India**	1.9	34.2	1998–9
**Nepal**	6.3	15.0	2001
**Bangladesh**	0.5	6.7	1999–2000

From: Family Planning Worldwide 2002 Data Sheet. Population Reference Bureau. Washington DC, June 2002.

Officers and program managers of national or international organizations involved with vasectomy in each country were contacted by e-mail beforehand in order to inform them about the purpose of the project and to seek their help in identifying (a) the most relevant individuals to meet, and (b) the most relevant vasectomy centers to visit. Based on this information, a field visit schedule was planned. The targeted international organizations were the United Nations Population Fund (UNFPA), Family Health International (FHI), EngenderHealth, International Planned Parenthood Federation (IPPF), Marie Stopes International (MSI), and Marie Stopes Clinic Society (MSCS). These organizations provide support to family planning programs in one or more of the selected Asian countries.

Data were collected between December 2003 and February 2004. In each country, 5 to 11 key informants such as vasectomy providers, directors of family planning clinics, national level family planning program managers, and/or managers of international organizations involved in family planning programs were interviewed. Information about the current situation with vasectomy, locally and in general in the country, and the interest and open-mindedness regarding the use of cautery and/or FI was gathered. Visits to 3 to 6 major vasectomy centers in urban and rural areas were done. Vasectomy techniques performed in each center visited were precisely described based on direct observation, when possible, using a data collection grid previously used in a FHI/EngenderHealth study [11,13]. In addition, with the consent of local authorities and the patients involved, vasectomy procedures performed by the providers were videotaped for further meticulous analyses.

Audio-visual material on vasectomy techniques using cautery and FI was presented and hands-on demonstrations were performed by one author (ML) according to the local situation and interest. ML has performed over 9000 vasectomies over the last 20 years, most using NSV combined with various occlusion techniques including cautery and FI. [1,15,24-26] Cautery handles and tips manufactured in USA (Advance Meditech International) and Canada (Walsh Medical Devices Inc.) were brought along in order to assess the feasibility of carrying out procedures under local conditions.

## Results

### Current state of vasectomy in visited countries

#### Cambodia

Vasectomy was almost non-existent in Cambodia until 2000. Since then RACHA (Reproductive and Child Health Alliance), a program initially managed by EngenderHealth which has grown into a Cambodian non governmental organisation (NGO), has promoted vasectomy in selected provinces in the countries. A total of 40 providers have been trained to perform both tubal ligation by minilaparotomy and no-scalpel vasectomy. In 2001, 2002, and 2003, 416, 199, and 281 vasectomies were performed, respectively [27]. These numbers, still very marginal, varied greatly in each center, and depended on the funding available to provide free access to vasectomy and to reimburse patients' travel expenses to the vasectomy center.

#### Thailand

In Thailand vasectomy is available mainly through PDA (Population and Community Development Association), a NGO providing and promoting male sterilization since 1976. PPAT (Planned Parenthood Association of Thailand), government hospitals, and private clinics also provide vasectomy services. Vasectomy incidence has steadily decreased over the past 20 years. At PDA, from a peak of 7,836 vasectomies performed in 1983, it felt to a low of 767 in 2003, a ten-fold decrease. [28] Since 2003, sterilization (male and female) is not subsidized anymore as national government program. National statistics on vasectomy were not available.

#### India

India possesses a structured and comprehensive national program promoting the use of no scalpel vasectomies (NSV). This program is funded by the United Nation Population Fund (UNFPA) with the Government of India providing centers for training and making available the necessary infrastructure at the training sites. As of December 2002, 309 NSV courses had been organized all across the country involving 51 states trainers, 58 district trainers, and 1,080 trainees. A total of 153,687 procedures were performed during these training sessions. [29] A national NSV meeting is organized on an annual basis by the NSV Surgeons of India . In 2003–04, 113,092 vasectomies were performed with an incidence of 0.043 new acceptors per 100 women of reproductive age. During the same period 4,873, 530 tubal ligations were performed.

#### Nepal

Providing vasectomy service to the population is embedded in the Nepal National Policy, Strategy, and Plans. Each year a projected number of cases is calculated for each district and region. Among the countries visited, Nepal has the highest incidence and prevalence of vasectomy. Nevertheless, the incidence has been decreasing over the recent years due to increased access to other contraceptive methods. In 2002–2003 surgeons performed 20,588 vasectomies with an incidence of 1.64 new acceptors per 100 women of reproductive age.[30]

#### Bangladesh

There is a national program supporting family planning including vasectomy services in Bangladesh. The use of vasectomy has been steadily decreasing from 151,125 vasectomies performed in 1985–86 to a low of 7,603 in 1996–97. [31,32] Since then numbers have slowly increased in the remaining of the decade to reach 43,203 in 2002–03 exceeding the number of tubal ligations (32,761).[31] Trends forecast an even higher number of vasectomies in 2003–04. However, the incidence of 0.12 new vasectomy acceptors per 100 women of reproductive age in 2002–03 remains relatively low.

### Vasectomy surgical techniques currently used in visited Asian countries

Overall 21 vasectomy centers were visited in five countries (Table 2). Almost all facilities were training centers and most vasectomies observed were performed by certified trainers or master trainers. In many centers, the first author (ML) assisted the surgeon and/or performed parts of NSV and occlusion of the vas using thermal cautery and/or FI.

**Table 2:** Vasectomy surgical techniques used in visited Asian countries

Centers Visited	Procedure observed	NSV	Cautery	Excision (cm)	Suture material	FI
**Cambodia**						
1	No	Yes	No	1–1.5	V 3-0	Yes
2	No	Yes	No	1	V 3-0	Yes
3	No	Yes	No	1	V 3-0	Yes
**Thailand**						
1	Video	Yes	No	2–3	S	Yes
2	Yes	±	No	1.5	S	±
3	Yes	Yes	No	1	S 3-0	Yes
**India**						
1	Yes	Yes	No	1	C 10	No
2	Yes	Yes	No	1	S 2-0	Yes
3	No	Yes	No	1	S 2-0	No
4	Yes	Yes	No	1	S 2-0	Yes
**Nepal**						
1	Yes	Yes	No	1	S 2-0	Yes
2	Yes	Yes	Electro	0.5–1	S 1-0	No
3	Yes	Yes	No	1	S 2-0	No
4	No	±	No	1–1.5	S 1-0	No
5	Video	Yes	Thermal	1	S 2-0	Yes
6	Yes	Yes	No	1	S 2-0	No
**Bangladesh**						
1	Yes	Yes	No	1	S 3-0	±
2	Yes	Yes	No	1	S 1-0	No
3	Yes	Yes	No	1	S 3-0	±
4	Yes	Yes	No	1	S 2-0	±
5	Yes	Yes	No	0.5	S 2-0	±

NSV = No Scalpel Vasectomy, FI = Fascial Interposition, V = Vicryl, S = Silk, C = Cotton.

#### Isolation of the vas

To isolate and expose the vas, NSV procedure combined with vasal block was performed in all centers visited. However, the technique was not equally mastered by all providers and trainers, revealing the needs for some training updates. In general the quality of the instruments was acceptable but in some centers performing NSV technique properly was limited by the use of inadequate oval-designed ring clamps or blunted dissecting forceps.

#### Occlusion of the vas

Simple ligation with suture material and excision of a small vas segment (LE) was performed in nearly all centers visited. There were two exceptions, both in Nepal. In one hospital-based center in addition to LE, the tips of the stumps were electro cauterized. In another, thermal cautery combined with FI interposition was used.

**Figure 1:**
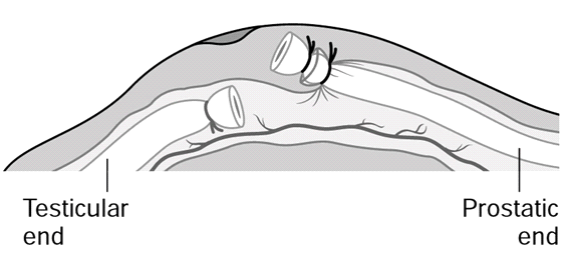
Vasectomy procedure using ligation and excision combined with fascial interposition over the testicular end.

The frequency of combined use of FI with LE varied from on country to the other. However, at all sites visited, nearly all surgeons were using or were taught the FI technique evaluated [10] and promoted [23] by EngenderHealth and FHI (Figure 1). This technique involves ligating the vas sheath around the abdominal stump thus covering the testicular end with the fascia. [23] In Cambodia where the vasectomy program is rather young, much emphasis has been put on performing FI along with LE. Although no cases were observed, it was said to be performed routinely in the three centers visited. In Thailand, the majority of vasectomy services are currently provided by PDA surgeons who routinely combine FI with LE. In the South Asian countries the situation was different. Although all trainers and providers met during the visit were aware of FI and the majority had some training in performing the technique, it was estimated that more than 95%, 97%, and 99% of vasectomies were done with simple LE without FI in India, Nepal, and Bangladesh, respectively.

There were many reasons reported for not performing FI. Firstly, this technique is difficult to master according to many trainers and providers. In a high number of procedures failed attempts to perform FI was observed. Training of providers may be insufficient as many trainers themselves do not routinely use the technique. One technical factor which may prevent surgeons from achieving proper FI was the size of the suture material. In Thailand where the technique was successfully performed routinely, Silk 3-0 was used to tie the vas. This fine thread would not interfere with the tied testicular stump of the cut vas sliding into its sheath when both stumps are returned back into the scrotum before pulling out the abdominal vas end to identify the vas sheath.[23] However, in South Asia many providers were using Silk 2-0, and even Silk 1-0 (Table 2). Even if available, most of the surgeons said they would be reluctant to use Silk 3-0 because such fine thread is believed to cut through the vas and to decrease the effectiveness of vasectomy. However, using larger size suture material may preclude from doing adequate FI.

Secondly, FI takes time. Even when well-mastered, performing FI may add 2 to 4 minutes to the LE procedure. Skilled NSV providers can complete a full LE vasectomy in about the same time. In high volume settings such as vasectomy mobile camps where several hundred men may be vasectomized each day, even trainers who teach FI do not do it because of the time constraints. Since a very high proportion of vasectomies are done in mobile camps in South Asia, the FI technique as now recommended may never be performed on a large scale for this reason.

Thirdly, the national standards of practice in the South Asian countries selected did not include FI as a mandatory step of vasectomy. There was no mention of FI in the Nepal standards[33], FI was optional ("preferable") in the Indian standards,[34] and although mandatory in the Bangladesh standards,[35] it was not specifically mentioned in the most recent national training manual[36].

There is a lack of data on the effectiveness and complication risks associated with the techniques currently used. A common belief is that the failure rate of vasectomy as currently performed is about 1% but one center reported a pregnancy rate as high as 4%. Semen analysis (SA) was available in some training centers but compliance was said to be low. All reported compliance under 30% except one center in Nepal reporting 90%. In this center, the failure rate based on repeat vasectomy was estimated to be 2 to 3%. Two centers had collected data on their failure rate. In Nepal, in a cohort of 644 vasectomized men using LE, vasectomy was repeated in 4 (1.6%) of the 263 men who had a SA performed. (Dr Kiran Shrestha, personal communication) In India, 3 (1.2%) pregnancies were encountered in 258 vasectomies performed with simple LE. In the same center, adding FI to LE resulted in no need to repeat vasectomy in 130 vasectomized men who all had at least one SA (Dr Kaur Baljit, personal communication). No data on complications were available but were said to be rare.

### Feasibility of introducing and evaluating cautery and FI in visited Asian countries

While FI was known by all vasectomy providers met, thermal cautery was new to most of them. Hand-held battery-driven thermal cautery devices specifically designed for vasectomy did not appear to be available in visited countries. About 20 vasectomies were performed involving local providers using the cautery devices brought from America. The technique used combined intraluminal thermal cautery and covering the prostatic end of the cut vas putting a free tie on the fascia. Beside the cautery device (handle and tips), all other material resources necessary to perform the technique including alkaline AA batteries were available in all countries visited, even in rural areas.

The repeated use of a thermal cautery device and tips was tested in a suburban mobile camp in Nepal and proved to be feasible. Reusable cotton sheaths for inserting the cautery handle were designed and made locally. They were autoclaved along with other sterile drapes. Cautery tips were decontaminated, brushed, washed, and processed with high level disinfection. [37]

In general infection prevention procedures were adequate for vasectomy except in some rural areas where, among other pitfalls, the surgical instruments were left in open air for many hours. Many providers are performing minilaparotomy for tubal ligation in addition to vasectomy, maintaining the same infection prevention standards for both procedures. With few exceptions, vasectomy surgical equipment was well maintained suggesting that cautery devices (handles and tips) could be kept functional with proper instructions and minimal training.

**Figure 2:**
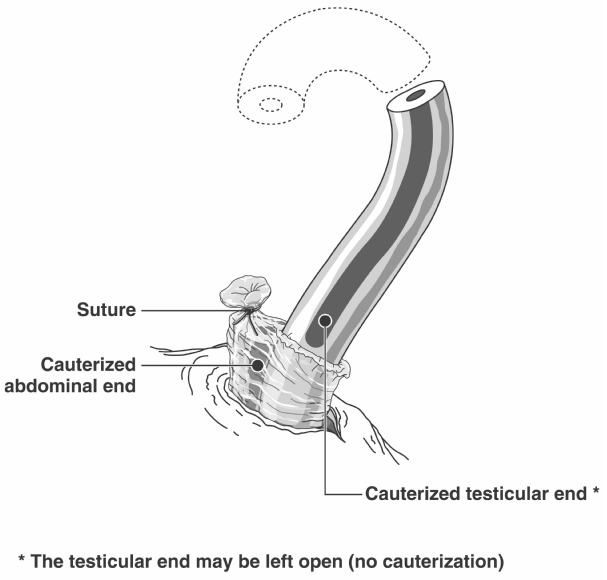
Vasectomy procedure with thermal cautery combined with fascial interposition over the abdominal end.

Providers demonstrated much interest in learning the use of cautery and FI as described in Figure 2. At least 10 trainers or master trainers performed vasectomy using the device. After reviewing the technique on video and observing one live case, all were able to demonstrate adequate use of the cautery device and FI under supervision. All considered that the technique was easy to learn, to master, and to teach. In the hands of skilled NSV providers, time to perform thermal cautery with FI for the first time was similar to that observed with current LE and FI technique. However, it definitely took more time on average than performing simple LE.

Cautery was already recommended as an optional occlusion technique in the Nepal standards of practice [33] but was not in India[34] and Bangladesh[35].

There was much interest to participate in the evaluation of efficacy and possible implementation of cautery on the part of leaders of the reproductive health/vasectomy programs and the service providers in South Asian countries. Potential structures to conduct evaluation research appeared to be in place in some locations. Some centers in Nepal had collaborated in international studies. Data were already collected in a structured format in all centers although with regional variations. Centers in India were using extensive data collection forms. Some centers in Nepal and India were already producing their own statistics. However, in most centers reinforcement of structures would be needed to improve the process of data collection and the validity of data collected.

Facilitating factors and barriers to the introduction and evaluation of cautery and FI in Asia are summarized in Table 3 and 4.

**Table 3:** Facilitating factors and barriers to the implementation of fascial interposition in Asia

**Fascial Interposition**		
	**Facilitating factors**	**Barriers**

**Technical aspects**	FI already implemented although not generalized	FI is difficult to learn and to master
		Use of FI difficult to implement in high volume settings because of time required to perform. Not a mandatory step in the national standard, and training protocol
**Human resources**	Interest in learning a new technique	Belief that current techniques are effective
	Interest in improving efficacy and decreasing complications	Changing current behavior
**Training**	Training infrastructures already in place in South Asia	Training new providers may take more time than training with simple LE
		Need to retrain existing providers
		Need to retrain surgical assistants
**Supplies**	No new supply needed (except extra suture material)	No supply of Silk 3-0 in national program
**Policy and program**	Program supporting sterilization (South Asia)	No program supporting sterilization (Thailand)
	FI already mentioned in some national standards of practice	FI not mandatory in most national standards of practice
**Evaluation**	Some infrastructure in place to conduct operational research	Low rates of follow-up and compliance to SA

**Table 4:** Facilitating factors and barriers to the implementation of thermal cautery in Asia

**Thermal Cautery**		
	**Facilitating factors**	**Barriers**

**Technical aspects**	Easiness to learn and to master thermal cautery	Need to modify FI technique when using cautery
	Thermal cautery may be used alone with probably better efficacy than simple LE	
	Cautery alone is faster to perform than any technique combined with FI	
**Human resources**	Interest in learning a new technique	Belief that current techniques are effective
	Interest in improving efficacy and decreasing complications	Changing current behavior
**Training**	Training infrastructures already in place in South Asia	Need to retrain existing vasectomy providers
		Need to train support staff (cautery device use and maintenance)
**Supplies**	"Low tech" supplies	Cost of new supplies (including batteries)
	Most supplies already in place	Thermal cautery devices not currently available
	Positive pilot field assessment of feasibility of processing and maintaining cautery devices	Processing and maintaining new material
	AA alkaline batteries readily available	
**Policy and program**	Program supporting sterilization (South Asia)	No program supporting sterilization (Thailand)
	Cautery included in some national standards of practice	Cautery not included in most national standards of practice
**Evaluation**	Some infrastructure in place to conduct operational research	Low rates of follow-up and compliance to SA

FI = Fascial Interposition, LE = Ligation and excision, SA = Semen Analysis.

## Discussion

The objectives of this project were to determine the extent of the vasectomy surgical techniques currently used in some Asian countries and to evaluate the feasibility of introducing and assessing the use of cautery and FI to occlude the vas in these countries. It was not possible within the limits of the project to do an exhaustive survey of all vasectomy techniques performed in South and South East Asia. However, we included two strategies that we believe were sufficient to achieve our objectives and to provide a sound basis for planning future operational research addressing the issues. First, in each country key-informants from various levels of the health care system related with male sterilization program were interviewed. To the exception of Thailand, this included the national authorities who are responsible for the vasectomy program, and who could provide an overview of the global situation in each country. Second, a convenience sample of 21 urban and rural vasectomy centers from various Asian countries was visited, including participation in daily clinical activities in most centers. Although these centers may not be fully representative of all vasectomy centers in Asia, there were very strong national standards regarding how family planning services must be provided in the countries visited. We thus expect much less variations in techniques used in the countries visited than in North American or European countries.

Most vasectomies in Asia were performed with NSV and simple LE. NSV is recognized as the best approach to expose the vas.[1] Based on current evidence if LE is used to occlude the vas, FI should also be performed to improve effectiveness of vasectomy.[1,10] This latter technique was largely known and even taught in the Asian countries visited but was seldom performed in South Asia countries (India, Nepal, and Bangladesh). The main reasons reported for not adopting the technique were: 1) insufficient surgical skills, 2) time needed to perform the technique, and 3) technique not being mandatory according to country standards. However, all through Asia, vasectomy program leaders were conscious of the importance of implementing FI as a routine procedure. As per example, FI was the theme of the 2^nd ^Indian National NSV Conference held in May 2004.

The use of a hand-held battery device thermal cautery for vas occlusion may be feasible in Asia. Timing for introducing cautery/FI would be right as training or retraining of vasectomy providers on combined use of FI and LE is currently needed in most Asian countries. Providers showed great interest in the use of the technique, but taking into account the fact that most were experienced trainers this may not necessarily reflect the views of the majority of providers. Pilot assessment on a small scale showed that the technique can be safely and effectively performed by Asian providers with human and material resources currently available.

On the other hand, it has to be kept in mind that the major benefit of introducing cautery with FI in Asia is related to the high occlusive and thus contraceptive effectiveness of the technique. Although, cautery combined with FI appears to be much more effective than LE combined with FI, firm and conclusive evidence of the superiority of one technique over the other are still lacking.[1] Moreover, introducing cautery with FI may be associated with the same implementation barriers encountered with introducing FI on a large scale. In addition, new direct costs (cautery devices and batteries) and indirect costs (training, processing, and maintenance of the devices) would have to be considered before implementing cautery on a large scale. PATH (Program for Appropriate Technology in Health) working in coordination with FHI and EngenderHealth has estimated that battery-driven cautery handles and tips could be manufactured at a very low price in Asia. Moreover, bench studies suggest that cautery handles and tips currently available in the United States and Canada are durable and can be safely reused (Dr D Sokal, personal communication).

## Conclusion

One of the characteristics of a successful vasectomy program in developing countries worldwide is the availability of skilled providers.[38] This means that providers must offer the most effective and the safest vasectomy method. Thermal cautery may prove to be this method. Further studies are needed however before thermal cautery is introduced in Asia on a large scale. These studies should assess effectiveness and surgical complications concomitantly with quantitative and qualitative outcomes related to the implementation of this new technique. Until thermal cautery is introduced in South and South East Asia, vasectomy providers should perform FI along with LE to maximize effectiveness of vasectomy procedure.

## Competing interests

The author(s) declare that they have no competing interests.

## Authors' contributions

ML, JP, and DS conceived of the study and participated to its design. ML, RK, SSB, MR, JB, GDB, TMV participated in the data collection. All authors provided input in the data analysis. ML drafted the manuscript and JP and DS revised the early versions. All authors read and approved the final manuscript.

## Pre-publication history

The pre-publication history for this paper can be accessed here:


